# Novel Green Biomimetic Approach for Synthesis of ZnO-Ag Nanocomposite; Antimicrobial Activity against Food-borne Pathogen, Biocompatibility and Solar Photocatalysis

**DOI:** 10.1038/s41598-019-44309-w

**Published:** 2019-06-05

**Authors:** Mina Zare, Keerthiraj Namratha, Saad Alghamdi, Yasser Hussein Eissa Mohammad, Abdo Hezam, Mohamad Zare, Qasem Ahmed Drmosh, Kullaiah Byrappa, Bananakere Nanjegowda Chandrashekar, Seeram Ramakrishna, Xiang Zhang

**Affiliations:** 1 Center for Materials Science and Technology, University of Mysore, 570006 Mysuru, India; 2 DOS in Earth Science, University of Mysore, 570006 Mysuru, India; 3 Laboratory Medicine Department, Faculty of Applied Medical Sciences, Umm Al-Qura University, Makkah, Saudi Arabia; 4 Department of Biochemistry, Applied Science college, Hajjah University, Hajjah, Yemen; 5 Health Science Center, Xi’an Jiaotong University, 710061 Shaanxi, China; 6 Physics Department and Centre of Research Excellence in Nanotechnology, King Fahd University of Petroleum and Minerals, 31261 Dhahran, Saudi Arabia; 7 Adichunchanagiri University, B.G. Nagara, Mandya District, 571448 Karnataka, India; 8 Laboratory of Advanced Materials Chemistry, Advanced Institute of Materials Science, Ton Duc Thang University, Ho Chi Minh City, Vietnam; 9 Faculty of Applied Sciences, Ton Duc Thang University, Ho Chi Minh City, Vietnam; 10 Department of Mechanical Engineering, National University of Singapore, 117576 Singapore, Singapore; 11 School of Mechanical Engineering, Beijing Institute of Technology, 100081 Beijing, China

**Keywords:** Nanoparticles, Nanoparticles, Synthesis and processing

## Abstract

A simple, eco-friendly, and biomimetic approach using *Thymus vulgaris* (*T*. *vulgaris*) leaf extract was developed for the formation of ZnO-Ag nanocomposites (NCs) without employing any stabilizer and a chemical surfactant. *T*. *vulgaris* leaf extract was used for the first time, in a novel approach, for green fabrication of ZnO-Ag NCs as a size based reducing agent via the hydrothermal method in a single step. Presence of phenols in *T*. *vulgaris* leaf extract has served as both reducing and capping agents that play a critical role in the production of ZnO-Ag NCs. The effect of silver nitrate concentration in the formation of ZnO-Ag NCs was studied. The *in-vitro* Antimicrobial activity of NCs displayed high antimicrobial potency on selective gram negative and positive foodborne pathogens. Antioxidant activity of ZnO-Ag NCs was evaluated via (2,2-diphenyl-1-picrylhydrazyl) DPPH method. Photocatalytic performance of ZnO-Ag NCs was appraised by degradation of phenol under natural sunlight, which exhibited efficient photocatalytic activity on phenol. Cytotoxicity of the NCs was evaluated using the haemolysis assay. Results of this study reveal that *T*. *vulgaris* leaf extract, containing phytochemicals, possess reducing property for ZnO-Ag NCs fabrication and the obtained ZnO-Ag NCs could be employed effectively for biological applications in food science. Therefore, the present study offers a promising way to achieve high-efficiency photocatalysis based on the hybrid structure of semiconductor/metal.

## Introduction

Metal nanomaterials and nanocomposites are attracting researchers across globe due to their superior magnetic, chemical, optical and electrical properties^1^. These properties of metal nanomaterials are remarkable in various application sections containing nonlinear optical machinery, nanoelectronical devices, catalysis, bio-medical, etc^2^. Metal oxides semiconductor nanocomposites have been widely studied due to their possible applications in various areas^3^.

In recent years, synthesis of metal NPs, by using green chemistry principles, are a possible alternative method to the routine physical and chemical methods, owing to very low cytotoxicity, greater film forming capacity and usage in a wide array of biomedical applications. Several studies have shown the synthesis of metal nanoparticles using plant, fungi, bacteria and other natural sources. However, using plants for nanoparticle synthesis might be advantageous over other biological processes because it eliminates the elaborate process of maintaining cell cultures, can be suitably scaled up for large-scale nanoparticle synthesis, and it is eco-compatible and cost-effective^4–6^. There have been several reports on photosynthesis of nanoparticles by employing leaf extracts of various plants including *Croton Caudatus Geisel*^7^, *Azadirachta indica* (Neem)^8^, *Nerium oleander*, *Coriandrum sativum* (coriander)^9^, *Phyllanthus amarus*^10^, and *Lawsonia inermis* (*henna*)^11^. In case of sundried *C*. *camphora* leaves, the polyol and water-soluble heterocyclic components were mainly found to be responsible for the reduction of silver or chloroaurate ions and stabilization of nanoparticles, respectively^9^. According to Kasthuri J., *et al*. the size and shape of the nanoparticles could be controlled by varying the concentration of the phyllanthin extract thereby to finetune the optical properties of the nanoparticles^10^.

Among metal oxides, ZnO-Ag NCs have attracted large attention, because of various desirable properties like ZnO has more chemical stability, low cost large surface area and wide-bandgap with several applications that include usage in solar cells, electronics, photoelectronics, and sensors, as Ag nanomaterials illustrate some distinctive properties in biological and chemical sensing and high electrical conductivity^12^. Additionally, Ag amendment is found to be effective for the synthesis of p-type ZnO, as the naturally occurring ZnO demonstrations n-type conductivity as a result of its innate defects such as oxygen vacancies and zinc interstitials^13^. Recently, Ag ions have gained more attention in several research studies due to their newly discovered effects on the evolution of antibacterial activity and efficiency of photocatalytic activity of semiconductor^14^.

On the other hand, recently ZnO nanoparticles (NPs) have attracted much consideration as it possesses a vast range of attributes based on doping, with a range of conductivity from insulating to metallic (consist of p-type and n-type conductivity), room-temperature ferromagnetism, piezoelectricity, large forbidden energy gap (3.37 eV at 300 K), high melting point ~1975 °C, greater transparency, wide binding energy (60 meV), semi-conductivity, chemical-sensing effects and huge magneto-optic^15^. ZnO NPs have been synthesized with numerous morphologies in the nanoregime, such as belts^15^, rods^16^, prisms^17^, flowers^18^, rings^19^ and many more. Various methods that have been used to fabricate ZnO-Ag NCs include hydrothermal synthesis^20,21^, template-confined synthesis routes^22^, Solvothermal method^23^ and microwave heating and sonochemical method^24^.

Recently, there has been an increased release of phenol and its derivatives, from various industries, such as agrochemical, petrochemical and pharmaceutical, into the ecosystem^25^. Most of these phenol derivatives, mainly 4- nitrophenol (4-NP) have been identified as toxic pollutants and can even be carcinogenic to human beings^26^. Thus the reduction reaction of toxic nitroaromatic compounds is of great significance. Among various semiconductors, ZnO is considered as more reliable material for pollutant removal due to its fast charge carrier mobility and prolonged electron life times in comparison to TiO_2_^27^. Noble metals like Au, Ag and Pt are one of potential choices to fabricate heterojunctions with enhanced photocatalytic performance for the degradation of nitrocompounds and other pollutants as per previous reports^28^. Lately, the researchers have claimed that the porous and open structure of ZnO favoured the effective inactivation by increasing the number of active sites and effective charge separation observed in the presence of Ag^29^. Ag acts as an electron sink and hole as hydroxyl radical (·OH) for the photochemical killing of bacteria. The scientists have proved that the photogenerated holes and ·OH are the main oxidative species for *Escherichia coli* (*E*. *coli*) inactivation^29^.

In the present article, ZnO-Ag NCs with 2 nm diameters of Ag particles via facile bio-hydrothermal method was reported. Silver nitrate and zinc nitrate as the source materials and sodium hydroxide as the precipitating agent were used. Impact of *T*. *vulgaris* leaf extract and incorporation of Ag into ZnO crystal on their structural, optical, and antibacterial behaviour, antioxidant activity, cytotoxicity and photocatalytic activity are studied in detail. The synthesized ZnO-Ag NCs exhibit excellent antimicrobial activity against selected gram-negative and gram-positive bacteria, antioxidant activity with less cytotoxicity and high photocatalytic activity under natural sunlight for the degradation of phenol.

## Results and Discussion

### Gas chromatography–mass spectrometry (GC-MS) analysis

**Figure 1:**
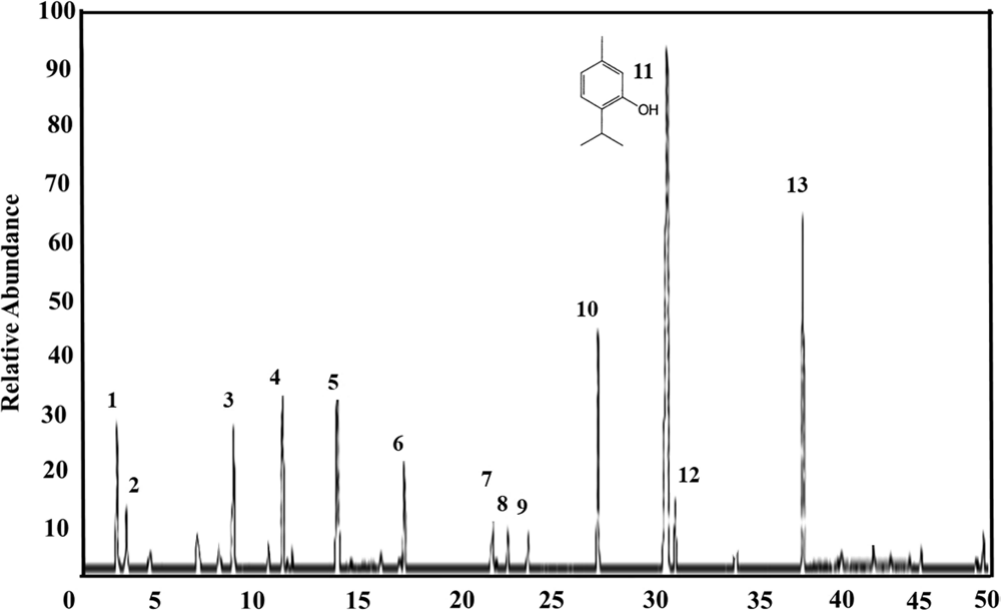
The typical TIC-GC/MS chromatograms of *T*. *vulgaris* leaf extract.

The GC-MS fingerprint profile of *T*. *vulgaris* was attained and shown in Fig. 1 and Table 1. The results demonstrate the presence of 13 major components (94.06%) in which phenols were the main compound in the extract. In general, plant extract is applied as a potential substitute for the reducing, capping and stabilizing agent due to the combination of various bio-substances such as alkaloids, terpenoids, tannins, phenolics, amino acids, saponins, proteins, polysaccharides, vitamins and enzymes^30,31^. Thymol is a type of phenolic monoterpenes which is the main chemical constituents in the leaf extract about 51.17% of *T*. *vulgaris*. Earlier reports have shown that flavonoids and phenols are involved in the stabilization, formation and bio-reduction of metal oxide and metal NPs^32^. Presence of OH groups in flavonoids and phenols are responsible for the reduction of zinc nitrate into ZnO NPs^33^. Various studies have reported that C=O–C, C=C and C=O groups in leaf extract may act as a stabilizer^34^. Flavonoids and phenols are present in in leaf extract of most medicinal plants and have been shown to be used as bio-reductants of metallic ions and display a wide range of biological activities^32,35^. The functional groups responsible for the capping, stabilizing and reducing agents were confirmed by Fourier transform infrared (FTIR) spectrum.

**Table 1:** Composition of *T*. *vulgaris* extract determined using GC-MS.

Sl. No.	Component	Retention index	Percentage (%)
1	α-Thujene	926	0.97
2	α-Pinene	932	0.08
3	Myrcene	993	0.84
4	α-Terpinene	1014	2.23
5	p-Cymene	1023	11.61
6	1,8-Cineo	1030	1.15
7	γ-Terpinene	1059	13.80
8	Linalool	1102	1.06
9	Borneol	1165	0.74
10	Carvacrol methyl ether	1246	2.44
11	Thymol	1292	51.17
12	Carvacrol	1300	4.00
13	β-Caryophyllene	1416	3.97
	Major Component		94.06
	Minor Component		5.94

### Characterization

**Figure 2:**
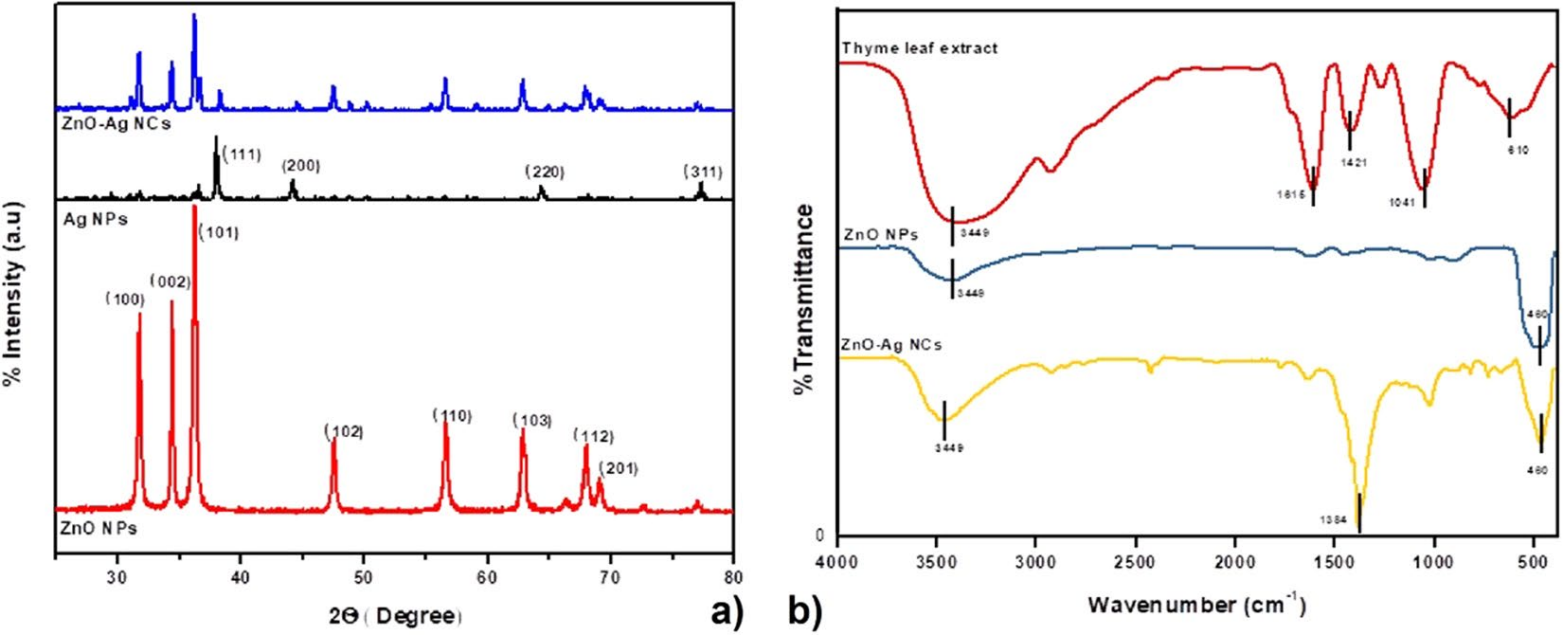
(**a**) XRD patterns of ZnO-Ag NCs, ZnO NPs and Ag NPs, (**b**) FTIR spectra of ZnO NPs ZnO-Ag NCs and *T*. *vulgaris* leaf extract.

X-ray diffraction (XRD) patterns of ZnO–Ag NCs were revealed by powder X-ray diffraction measurement. Figure 2(a) illustrates the XRD pattern of ZnO-Ag NCs, bare ZnO NPs and Ag NPs. The peaks in the XRD pattern show the face-center-cubic (fcc) metallic structure of Ag (JCPDS card no. 04-0783) and hexagonal wurtzite structure of ZnO (JCPDS card no. 36-1451). For Ag NPs, the main characteristic peaks at 2θ values 37.2, 44.6 and 64.5, which belonged to the (111), (200), (220) planes of fcc, approving the formation of Ag because of the substitution of Ag^+^ ions for Zn^2+^ in the ZnO lattice. The XRD peaks for ZnO-Ag demonstrate the formation of clear, distinct phases for both Ag and ZnO. Formation of distinct phases for both ZnO and Ag in the ZnO–Ag is reveals the synthesis of crystalline nanocomposite. The identified peaks represent the purity of nanocomposite^36^.

The typical IR spectrum of ZnO NPs, ZnO-Ag NCs and *T*. *vulgaris* leaf extract are given in Fig. 2(b). FTIR spectra of NCs exhibited prominent peaks at 460, 1385 and 3449 cm^−1^. The strong absorption peak at 460 cm^−1^ is attributed to Zn–O stretching vibration of the ZnO NPs^37^. After formation of Ag NPs on the surface of ZnO NPs, the intensity of ZnO peak was reduced. The spectral band at 1384 cm^−1^ corresponds to the symmetric stretching of acetate species^38^. The existence of broad band at 3449 cm^−1^ corresponds to the stretching vibration of the O–H mode. This may be owing to the OH groups of water^36^. The absorption peak in *T*. *vulgaris* leaf extract at 1041 is due to to C–N stretching in primary amine^39^. The small peak at 1615 cm^−1^ is ascribed to the O–H bending mode due to adsorption of water molecules^40^. *T*. *vulgaris* leaf extract possess polyphenolic compounds whose main components are thymol, isothymol, monoterpenes, linalool and α-terpnoeol, respectively^41^.

**Figure 3:**
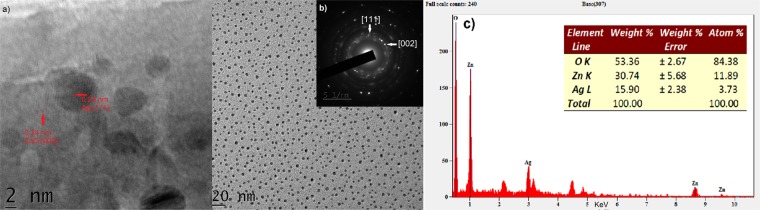
(**a**) HRTEM image display lattice fringes of both Ag and ZnO, (**b**) SAED pattern of ZnO-Ag NCs is inset of TEM image, (**c**) EDX spectrum of ZnO-Ag NCs.

Figure 3(a) depicts the morphology and size of synthesized ZnO-Ag NCs using transmission electron microscopy (TEM). The TEM images display the formation of ~5 nm spherical Ag NPs on the surface of ZnO particles. NCs were very obvious from TEM images. Figure 3(b) illustrates the corresponding selected area electron diffraction (SAED) pattern of ZnO-Ag NCs. The high resolution TEM result displays the fringes of 0.26 and 0.235, which are corresponding to [002] and [111] crystal planes of ZnO and Ag, respectively. This is in agreement with SAED and XRD pattern. The crystalline nature of ZnO- Ag NCs was proved by the SAED pattern with the observation of bright spots. The appeared bright spots are attributed to (100), (102) and (101) plane of hexagonal structured ZnO while the mild spots are due to (111) and (200) plane values of Ag crystal. Figure 3(c) demonstrates the corresponding energy dispersive X-ray spectrometer (EDX) pattern. The result displays there aren’t other impurities in EDX profile. The EDX findings visibly signify the presence of Zn, Ag and O in the synthesized NCs. The elemental analysis by EDX represents 30.74, 15.90 and 53.36 weight percentage of NCs is for Zn, Ag and O respectively.

**Figure 4:**
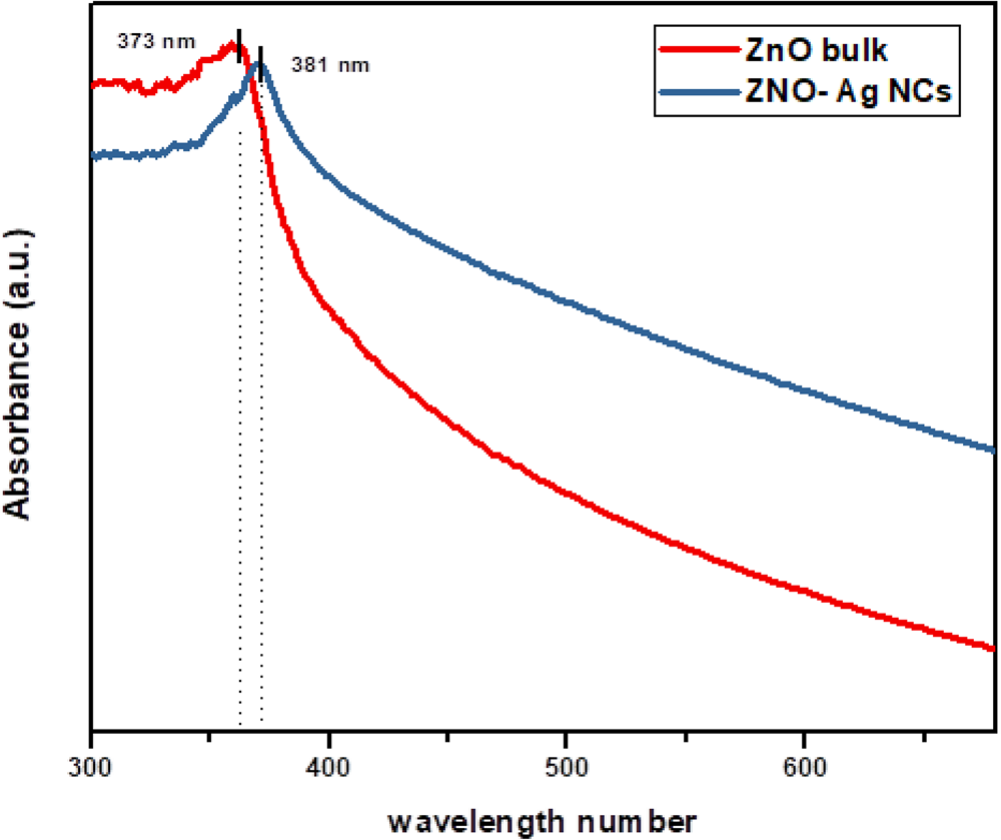
UV*–*Vis absorption spectrum of ZnO-Ag NCs and bulk ZnO.

The optical absorption of ZnO-Ag NCs at ambient temperature was evaluated using spectrophotometer. Figure 4 shows the UV- visible spectroscopy of prepared samples and pristine ZnO. The UV*–*Vis spectrum of ZnO-Ag NCs and bulk ZnO displayed a maximum absorption peak at 381 and 373 nm, respectively. The band gap of bulk ZnO and ZnO-Ag NCs was found to be 3.32 eV and 3.25 eV, respectively. The presence of Ag NPs improves the band gap absorption in comparison to the bare ZnO NPs^29^. Ag NPs function ranges in between the conduction band (CB) and valence band (VB) of ZnO NPs that accelerates the light absorption capacity^42^.

**Figure 5:**
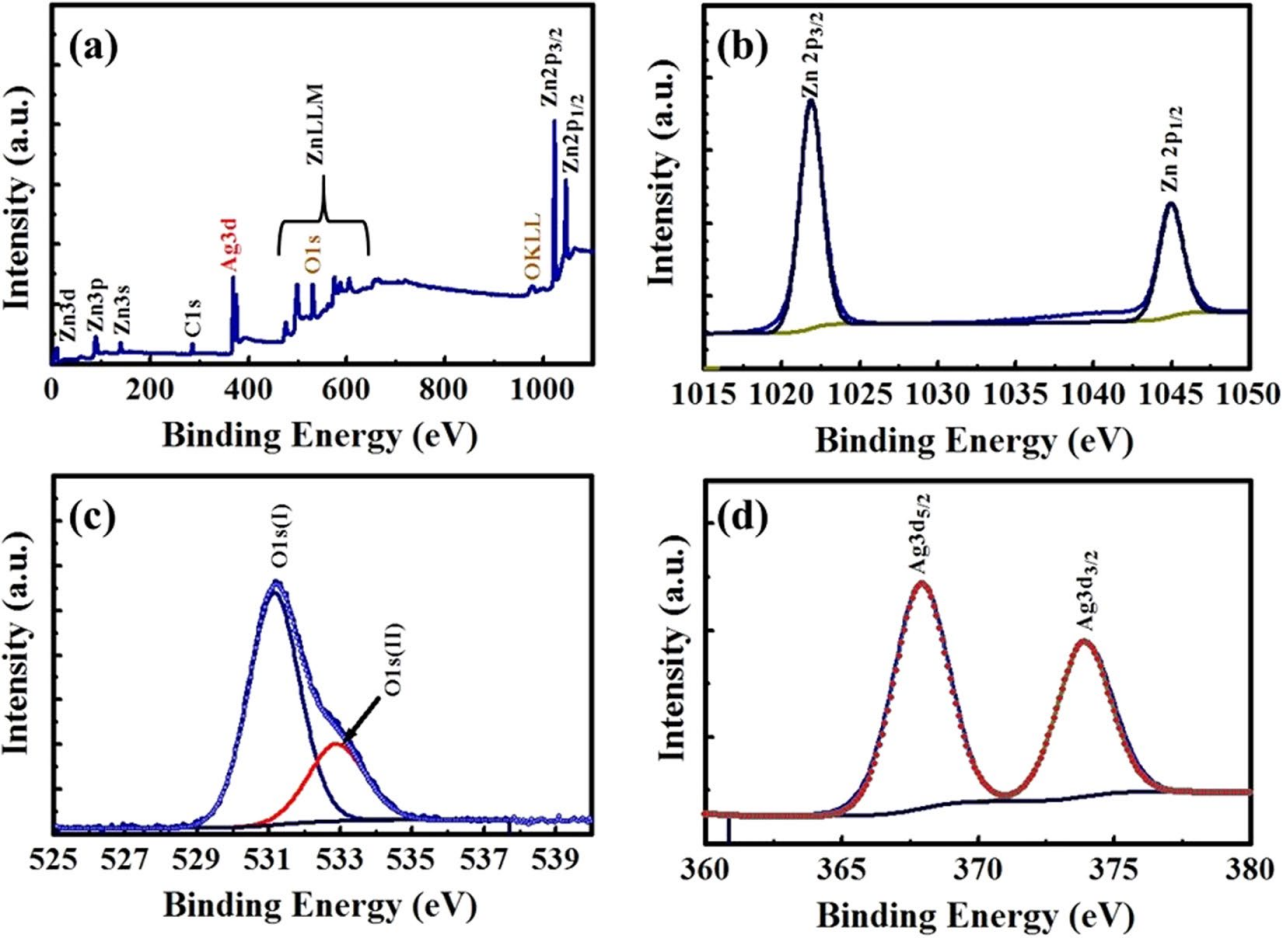
XPS analysis of ZnO-Ag NC sample. (**a**) XPS survey spectrum, and the core level XPS analysis of (**b**) Zn2p, (**c**) O1s, and (**d**) Ag3d.

In order to investigate the surface electronic structure and composition of ZnO-Ag NC, X-ray photoelectron spectroscopy (XPS) measurements were employed. The survey XPS spectrum of the ZnO-Ag NC is shown in Fig. 5, which reveals that the sample contains only Zn, O, C, and Ag. Figure 5(b–d) show the high resolution XPS spectra of the Zn2p, O1s, and Ag3d core levels, respectively. The Gaussian-resolved result for the Zn2p spectra in Fig. 5(b) shows two peaks centred at 1021.86 eV and 1044.98 eV, which were assigned to Zn 2p3/2 and Zn 2p1/2, respectively. The spin orbit splitting of 32.12 eV between these two peaks are consistent with that reported for photoelectrons excited from Zn^+2^ ions in the ZnO crystal lattice. The O1s core level spectrum illustrated in Fig. 5(c) can be deconvoluted into two peaks (O1s (I) and (O1s(II)). The peak at 531.23 eV is ascribed to oxygen anions in the ZnO crystal lattice. While the peak at 533.13 eV may be assigned to OH group absorbed onto the surface of the ZnO-Ag. Figure 5(d) displays a high resolution XPS spectrum of Ag 3d in ZnO-Ag NC. Two obvious peaks at about 367.91 eV and 373.88 eV corresponded to Ag 3d_5/2_ and Ag 3d_3/2_, respectively. These binding energies values and XRD results unambiguously confirm that Ag is present only in the metallic form.

### Antimicrobial activity

**Figure 6:**
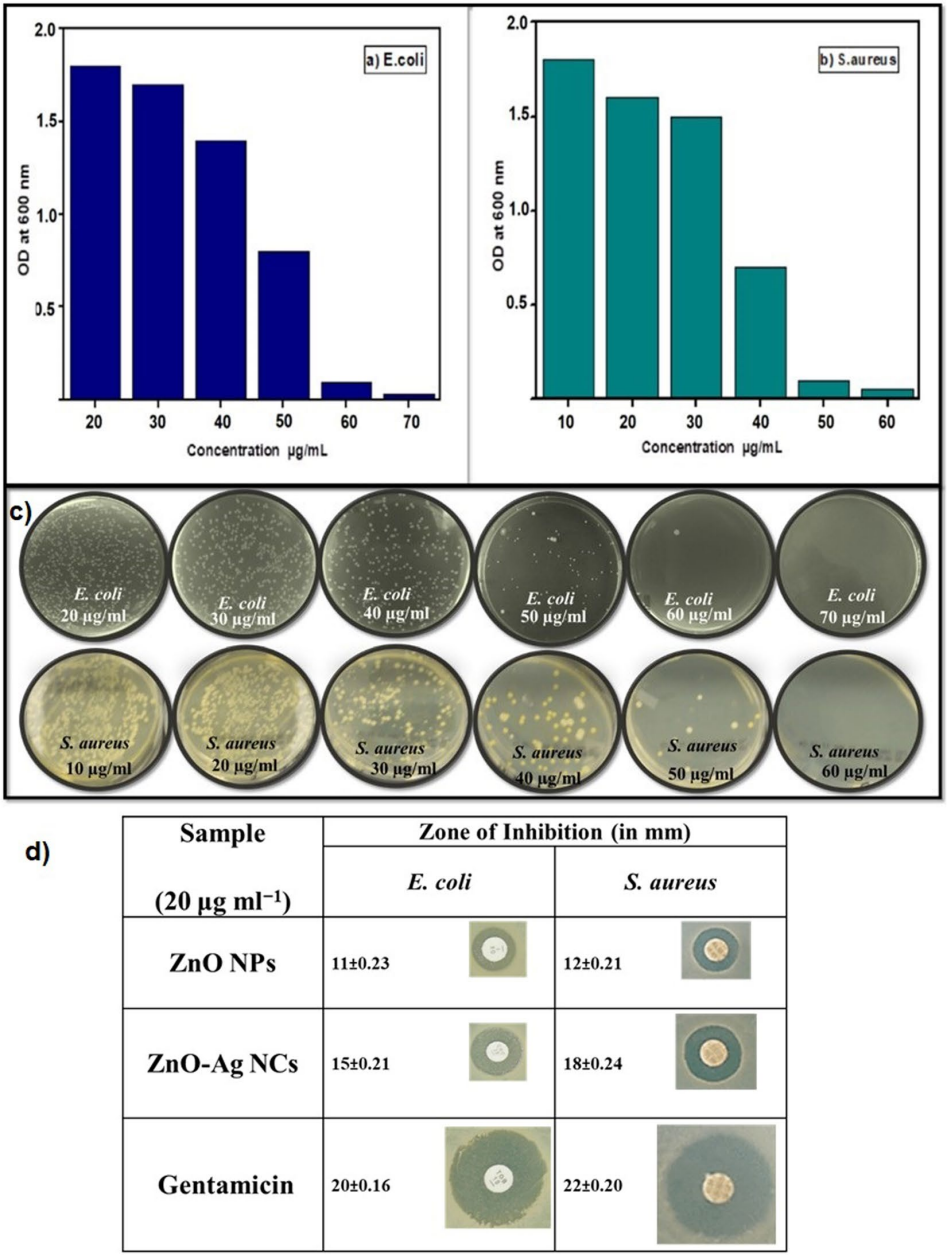
Effect of different concentrations of Ag–ZnO NCs on growth of (**a**) *E*. *coli*, (**b**) *S*. *aureus* and (**c**) growth of bacterial colonies on agar plates, (**d**) Inhibition Zone of ZnO-Ag NCs, ZnO NPS and Gentamicin.

The aim of this study is to evaluate the inorganic materials (ZnO-Ag NCs) for antibacterial potency in the water treatment for safe drinking. The proposed mechanisms of antibacterial activity of nanomaterials can be classified mainly into two categories: (1) create secondary products that cause cell injury, (2) physically interact with the bacterial cells (e.g. disrupting/penetrating the cell wall and oxidizing cell components)^19^. Antimicrobial effect of ZnO and Ag on the microbial cell is obvious but only a few studies are presented in the case of antimicrobial potency of doped ZnO^19^. According to Ghosh *et al*., the potential mechanism for inhibiting growth of bacteria is due to the physical interaction of doped ZnO NPs with the bacteria^13^. This study was carried out using selective food-borne pathogens *Staphylococcus aureus* (*S*. *aureus*) and *E*. *coli*. Figure 6 exhibits the effect of different concentrations of ZnO-Ag NCs on growth of (a) *E*. *coli*, (b) *S*. *aureus* and (c) growth of bacterial colonies on agar plates. Minimum Inhibitory Concentration (MIC) and Minimum Killing Concentration (MKC) values for the ZnO-Ag NCs were determined by observing the growth of *S*. *aureus* and *E*. *coli*. Visual turbidity analysis displayed that 50 and 60 µg/ml concentration of the ZnO-Ag NCs inhibited the growth of *S*. *aureus* and *E*. *coli* and they were considered as MIC values, respectively that is less than earlier reported^43,44^. Then re-inoculation into fresh Luria broth medium with a various concentration of ZnO-Ag NCs, no growth was detected for the nanocomposite with 60 and 70 µg/ml for *S*. *aureus* and *E*. *coli* respectively, which were considered as MKC value. This study clearly proposes a strong antibacterial potential of ZnO-Ag NCs. Hence, our green synthesized nanocomposite can be utilized as a reliable and more effective nano-weapon against food-borne bacteria for water purification and food packaging applications.

### Disc diffusion assay

Disc diffusion method was executed to evaluate qualitative assessment of antimicrobial activity. Inhibition zone diameter values were measured and comparative study of the inhibition zones was made. Remarkably, the ZnO-Ag NCs displayed lesser effect for *E*. *coli* than *S*. *aureus*. This may be because of the fact that gram-negative bacteria are less susceptible to antibacterial potency than gram-positive bacteria, caused by the impermeable and thinner peptidoglycan layer^13,45–47^. Antibacterial potency of ZnO-Ag NCS against microorganisms depends on membrane structure integrity or cell wall integrity^48^. Also, - antibacterial activity of ZnO-Ag NCs is greater than ZnO NPs is because of the strong interaction among semiconductor ZnO and metallic Ag for both gram-negative and gram-positive bacteria^13^. Figure 6(d) indicates the activities of the ZnO-Ag NCs, ZnO NPS and Gentamicin. From the results, one can noticeably suggest that ZnO-Ag NCs is an effective antimicrobial agent for the selected bacteria. Each experiment was carried out in three sets and the outcomes were displayed as the mean of the triplicates with the standard deviation range. Standard error (SE) of mean values were calculated for the data.

### *In-vitro* antioxidant activity

An antioxidant is a substance that protects cells against the damaging influence of Reactive oxygen species (ROS)^49^. The DPPH assay is a quick, simple and sensitive method for the antioxidant screening of nanomaterials^40^. Molecules of antioxidant property possess extensive industrial and biomedical uses. Free radicals are neutralized by scavenger materials in the body^39^. The pharmacological role of nanomaterials is evaluated by *in-vitro* antioxidant activities. Two *in-vitro* total antioxidant capacity (TAC) techniques are Electron transfer approach (ET) and hydrogen atom transfer approach (HAT)^50^. In DPPH method, which is an ET technique, free radicals are quenched by antioxidants and alter the color upon reduction. DPPH have an unpaired electron in nitrogen atom and π system^51^. With altercation on aromatic ring, molar absorptivity is increased therefore, the substituent extend conjugation size. Eventually, the prolonged conjugation usually affect moves in the benzene absorption bands. The maximum peak at 517 nm is responsible for n → π * energy transition^52^. According to following HAT mechanism the reaction equation is formulated:

1 $${\rm{DPPH}}+{\rm{AOH}}\to {{\rm{DPPH}}}_{{\rm{2}}}+{\rm{AO}}$$

where DPPH with max = 517 nm is a stable chromogen radical.

**Figure 7:**
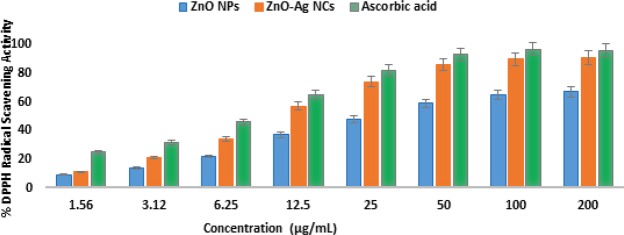
Percentage DPPH radical scavenging activity of ZnO-Ag NCs, ZnO NPs, and Ascorbic acid.

With an increase in the amount of ZnO-Ag NCs, the antioxidant activity is increased. The scavenging potency of samples were measured 91.42% and 65.66% at the highest amount of 100 µg/ml for ZnO-Ag NCs and ZnO NPs. The scavenging potency of ZnO-Ag NCs, ZnO NPs and control is displayed in Fig. 7 and it is obvious that the scavenging potency of ZnO-Ag NCs is increasing with increment in the quantity. The obtained ZnO-Ag NCs confirmed the increasing order of their scavenging potency. The incorporation of Ag into ZnO NPs significantly improves their oxidation capabilities^49^. This can be due to the synergetic effect of the nanocomposite. This represents that the synthesized ZnO-Ag NCs possess greater antioxidant activity in terms of scavenging DPPH free radicals. There are numerous studies of intrinsic scavenging potency for ZnO and Ag NPs. Therefore, we presume that the combined impact of ZnO and Ag NPs has revealed great scavenging potency.

IC_50_ value assists in the determination of substances quantity able to prevent 50% of used DPPH. Lesser the IC_50_, greater is the scavenging potency. The ZnO-Ag NCS presented an IC_50_ value of ~10.3 µg/mL, while ascorbic acid requires lesser amount of ~7.02 µg/mL and ZnO NPs showed an IC_50_ value of ~30.17 µg/ml by which it can be inferred that the ZnO-Ag NCs had a considerably comparable scavenging potency and IC_50_ value to that of standard ascorbic acid.

### *In-vitro* cytotoxicity (haemolysis assay)

**Figure 8:**
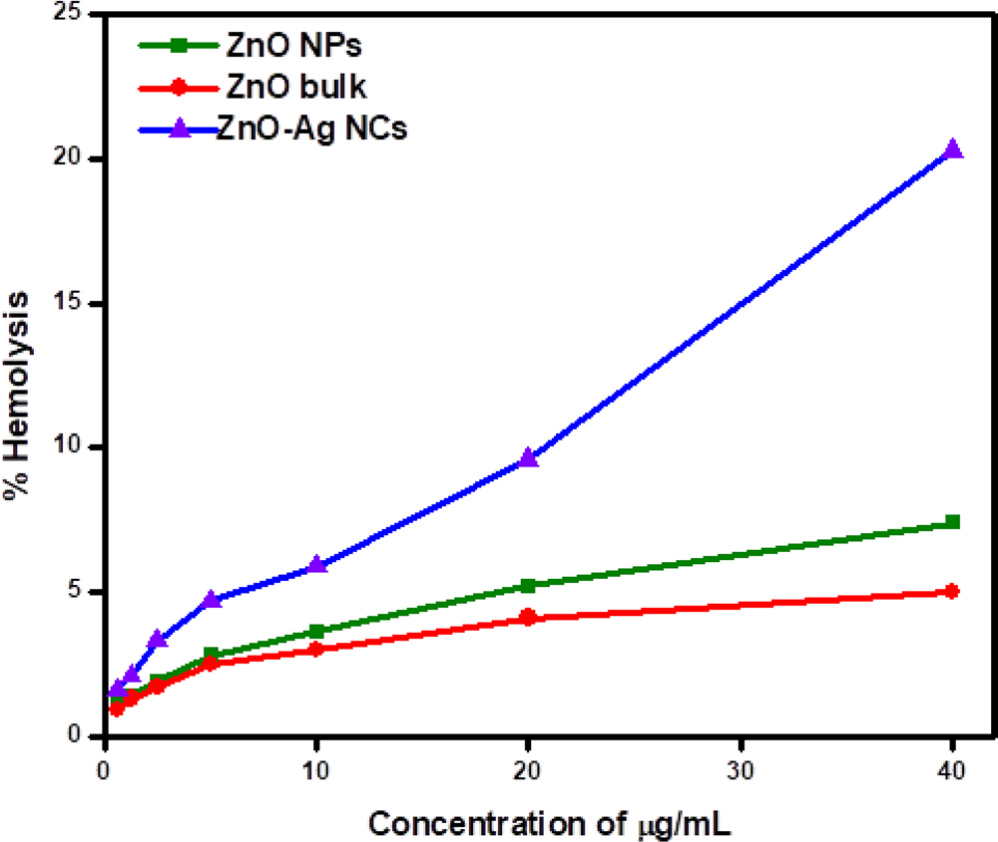
Percentage haemolysis of synthesized ZnO-Ag NCs, ZnO NPs and ZnO bulk.

Figure 8 depicts the percent haemolysis of RBCs after exposure to nanomaterials for 2 hours. It was obvious that ZnO-Ag NCs caused the visible release of hemoglobin from injured RBCs at lesser NPs exposure quantity. This result revealed that ZnO-Ag NCs possess higher hemolytic activity than ZnO NPs. ZnO-Ag NCs caused the considerable higher amount of haemolysis, beyond 7% haemolysis at 10 µg/mL. In comparison, ZnO NPs and bulk ZnO poses less than 8% haemolysis even at the upper quantity. ASTM E2524-08 standard described that % haemolysis more than 5% shows that the NPs effects harm to RBCs^53^. This rate was exceeded at the amount of 5, 20 and 40 µg/mL for ZnO-Ag NCs, ZnO NPs and bulk ZnO, respectively.

### Photocatalytic activity

**Figure 9:**
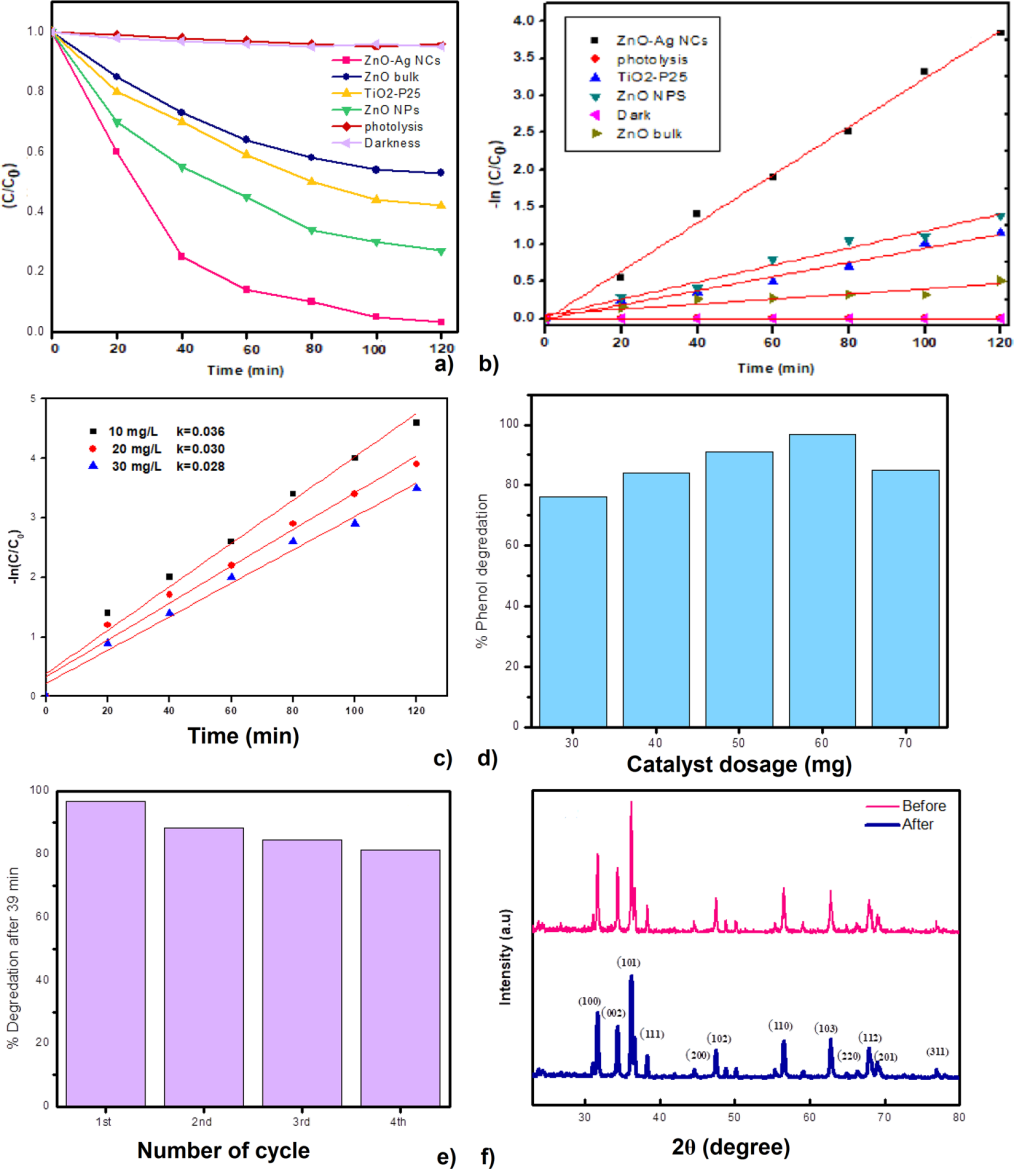
(**a**) Degradation curves. (**b**) Degradation kinetics of ZnO-Ag NCs, ZnO NPs, ZnO Bulk and TiO_2_-P25 under sunlight irradiation (catalyst amount = 60 mg, phenol initial concentration = 20 mg/L). (**c**) The impact of initial amount of phenol on photocatalytic activity. (**d**) The impact of photocatalyst dose on the elimination efficacy of phenol. (**e**) Cycling times of the photodegrading phenol applying ZnO-Ag NCs catalyst. (**f**) XRD patterns of ZnO-Ag NCs after and before 4 repetition.

Sunlight photocatalytic performance of organic hazardous compounds is encouraging for water treatment approach. Photocatalytic degradation of NCs were carried out using phenol as an organic pollutant, under sunlight irradiation. Phenol is an organic pollutant model without color and has no absorption in the range of 400–700 nm. 60 mg catalyst and a higher concentration of phenol, 20 mg/L was used for the photocatalytic activity. Figure 9(a) illustrates the time-dependent decomposition of phenol using ZnO-Ag NCs catalyst. It has been confirmed sample as prepared above leads to a remarkable enhancement in the photocatalytic degradation, even better than TiO_2_-P25. The phenol concentration has no change in the absence of photocatalyst (photolysis), visible light irradiation (darkness) and under sunlight irradiation for 120 min.

These results demonstrate that the photolysis and adsorption of phenol can be ignored in comparison with ZnO-Ag NCs. Remarkably, NCs illustrate the maximum photocatalytic activity (97.2% in 120 min) in comparison with ZnO NPS (86.5%), TiO_2_-P25 (64.8%) and bulk ZnO (43%) for phenol degradation due to their narrow forbidden energy gap. Fig. 9(b) and Table 2 display the rate of K constant values which were attained by using the slopes of –ln(C/C_0_) versus t. The result shows k value for ZnO-Ag NCs was nearly 3.5 and 3 times greater than TiO2-P25 and ZnO NPs, respectively. These results clearly advocate that photocatalytic degradation potential of ZnO-Ag NCs is considerably enhanced.

**Table 2:** Slopes of –ln(C/C0) versus t of the various photocatalysts for the degradation of phenol under visible light irradiation.

Equation	y = a + b*x	Value	Standard Error	Adj. R-Square
ZnO-Ag NCs	Intercept	−0.01231	0.05934	0.99616
ZnO-Ag NCs	Slope	0.03246	8.23E-04	
ZnO NPS	Intercept	0.01601	0.03775	0.9875
ZnO NPS	Slope	0.01141	5.24E-04	
TiO2-P25	Intercept	−0.00557	0.04055	0.9793
TiO2-P25	Slope	0.00949	5.62E-04	
ZnO bulk	Intercept	0.05464	0.03909	0.86792
ZnO bulk	Slope	0.00345	5.42E-04	

### Impact of phenol initial concentration

The primary amount of phenol shows a crucial improvement in water purification procedure. Figure 9(c) indicates the impact of phenol amount on photocatalytic performance. The degradation of dye reduces in the presence of a high volume of phenol due to the fact that higher amount of phenol molecules inhibits sunlight absorption by the ZnO-Ag NCs. Consequently, the amount of •OH and super oxide radicals (•O^2−^) forming on the surface of catalyst decreases, therefore the degradation efficiency declines.

### Effect of photocatalyst dosage

The impact of the catalyst dosage on the photocatalytic activity of phenol has been determined using various photocatalyst concentrations (30 to 70 mg/L). Figure 9(d) displays the impact of photocatalyst dose. The primary concentration of organic pollutant was 30 mg/L. The organic pollutant elimination increases to a highest level of 97.2% when the photocatalyst dosage is incremented to 60 mg, because of the increase in the nanoparticles density in the illumination area^54^. Additional increment in the photocatalyst dose beyond 60 mg declines the elimination efficacy of phenol because of light scattering by extra photocatalyst nanoparticles in the aqueous solution^55^. Therefore, the optimum photocatalyst amount of ZnO-Ag NCs is 60 mg.

### Reusability of photocatalyst

The reusability of ZnO-Ag NCs as a catalyst was executed using dye decomposition method under sunlight. The photocatalyst nanocomposites were centrifuged, washed and dried systematically before applying for next time. The photodegradation assessments were carried out for four cycles by sunlight and the photodegradation outcomes depict 96.8%, 88.1%, 84.5% and 81.3% of dye degradation for 1^st^, 2^nd^, 3^rd^ and 4^th^ cycles, respectively. Figure 9(e) illustrates the results of phenol degradation after 20 min under sunlight irradiation for four cycles. ZnO-Ag NCs displays remarkable photostability even after 2^nd^ cycle. There is no remarkable alteration in the photodegradation efficiency of ZnO-Ag NCs after the 2^nd^ time. The crystal structure constancy of the ZnO-Ag NCs was confirmed with the help of XRD as illustrated in Fig. 9(f). The results exhibit that the crystalline structure of applied ZnO-AG NCs catalyst did not change and confirm its activeness during photodegradation procedure. Hence ZnO-Ag NCs can be apply for wastewater purification.

### Mechanism of the photocatalytic degradation of phenol

**Figure 10:**
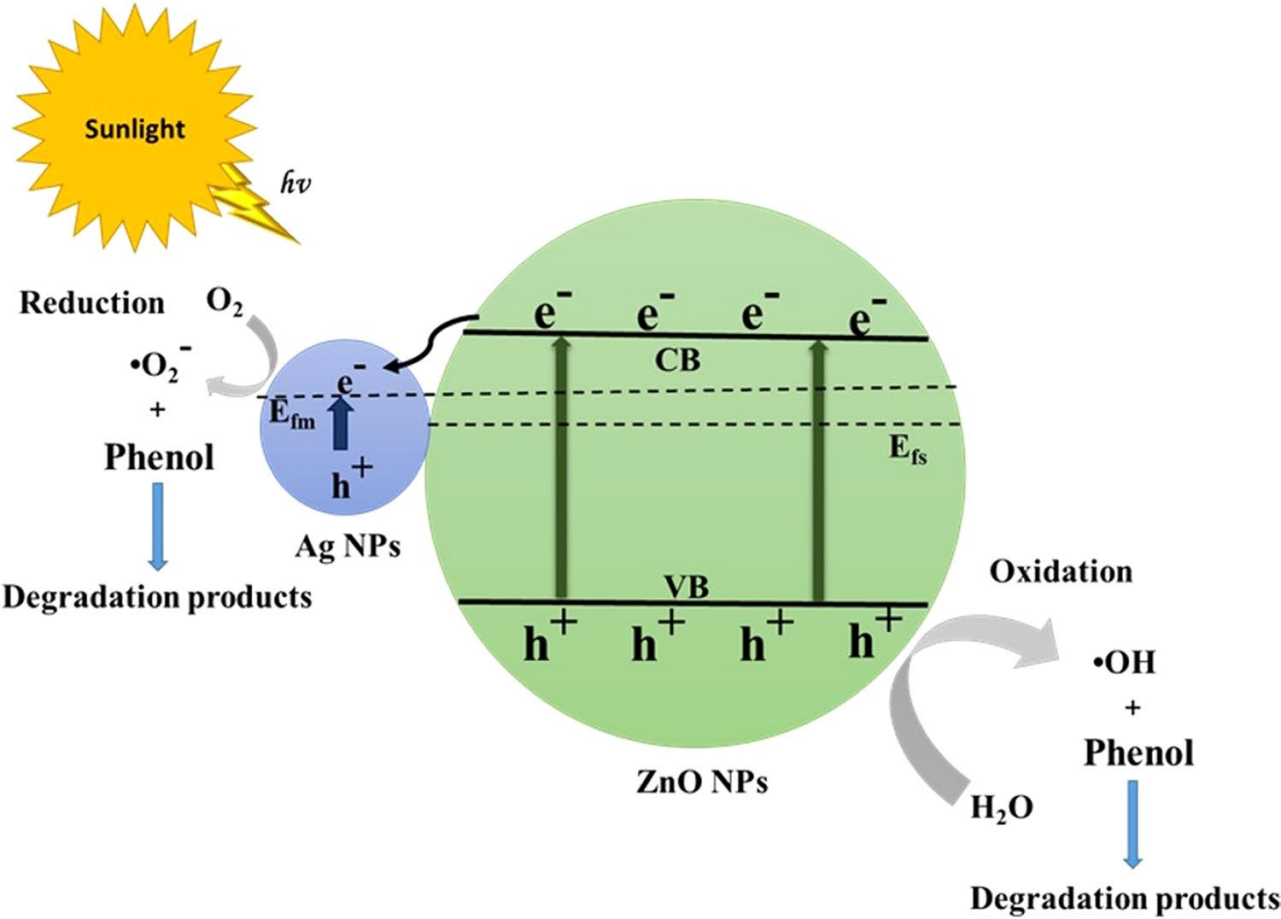
Schematic diagram showing the proposed photocatalytic mechanism of the Ag–ZnO NCs.

The enhanced photocatalytic performance of Ag–ZnO nanoparticles can be explained as follows. When the ZnO nanoparticles absorb photons of energy greater than or equal to its band gap energy, electrons and holes are generated in conduction band (CB) and valence band (VB), respectively. Since the energy level of the ZnO CB is higher than the Fermi level (E_fm_) of Ag–ZnO, electrons can transfer from ZnO to Ag nanoparticles. Hence Ag nanoparticles can trap the photoinduced electrons, inhibiting their recombination with holes. Photoinduced electrons can produce •O_2_ while holes in the ZnO VB and can react with H_2_O to produce •OH, both of which cause phenol degradation. The proposed mechanism is presented in Fig. 10.

$$\begin{array}{rcl}{\rm{ZnO}}+hv & \to  & {e}^{-}({\rm{CB}})+{h}^{+}({\rm{VB}})\\ {{\rm{e}}}^{-}\,+\,{{\rm{O}}}_{2} & \to  & \bullet {{\rm{O}}}_{2}^{-}\\ {{\rm{Ag}}}^{+}+{{\rm{e}}}^{-}({\rm{CB}}) & \to  & {\rm{Ag}}\\ {{\rm{h}}}^{+}+{{\rm{OH}}}^{-} & \to  & \bullet {\rm{OH}}\\ \bullet {\rm{OH}}+{\rm{Organic}}\,{\rm{dye}} & \to  & {\rm{Degradation}}\,{\rm{products}}\end{array}$$

Thus, the enhanced photocatalytic activity of Ag-ZnO can be attributed to formation of the Schottky junction at the Ag–ZnO interface which leads to improve the charge carriers separation and hence decrease their recombination rate.

## Methods and Materials

### Materials

*T*. *vulgaris* leaf was picked up from Nandi hills attached to Chamundi hill,  Mysuru, India. The anlytical grade of all chemicals was used in the preparation of samples in this work and was used as received. Silver nitrate (N/10, Rankem, India), NaOH (99%, Sigma-Aldrich, India), Zn (NO_3_)_2_·6H_2_O (99%, Alfa Aesar, India), ethanol (99%, Alfa Aesar, India), physiological saline (0.85%, Nicechem. India), Dulbecco’s phosphate buffered saline (DPBS) (Hi-Media, Inida), Degussa TiO_2_-P25 (20% rutile, 80% anatase with a mean nanoparticle dimension of 20 nm and a BET surface area of 35–65 m2 g-1), phenol (Sigma Aldrich, >99%), Luria broth (Hi-media, India), nutrient agar (Hi-media, India), DPPH (Hi-Media, India) were purchased. *S*. *aureus* (MTCC6908) and *E*. *coli* (MTCC 1698) from MTCC, Chandigarh, India were procured. Deionized water (DI water, ELGA, PURELAB Option Q7, 18.2 MΩ cm) was used during the studies.

### Preparation of *T*. *vulgaris* leaf extract

*T*. *vulgaris* leaf was collected and washed with double distilled water and dried in a hot air oven for 3 days at 50 °C. Then, fine powder of *T*. *vulgaris* leaf was prepared by grinding in the mixture. Next, 4 g of obtained *T*. *vulgaris* powder was mixed in 20 ml double distilled water and stirred for 30 minutes at 60 °C. The leaf extract sample was centrifuged for 10 minutes at ambient temperature. The upper layer was filtered with filter paper (11 μm Particle retention)^56^. The 20% final extract of the leaf was used for the fabrication of ZnO-Ag NCs.

## Methods

The synthesis of ZnO NPs with *T*. *vulgaris* extract was shown in our previous study^12^. ZnO-Ag NCs was manufactured with slight modification involving single step bio-hydrothermal synthesis. In this study, the best concentration of previously reported articles^12^ was considered as a basic and silver nitrate volume was altered to synthesize ZnO-Ag NCs. General purpose autoclaves were applied for the preparation of ZnO-Ag NCs. Zinc nitrate (0.5 M), Silver nitrate (10/N), and sodium hydroxide (10 M) solution with different volume were prepared and mixed under the stirring condition for 30 minutes, then *T*. *vulgaris* leaf extract was added to above solutions and after that sonicated for 30 minutes. The samples were shifted to the teflon liner and kept in an autoclaved vial in a hot air oven at 180 °C for 3 hours. Afterwards the samples were cooled down to ambient temperature and washed and dried. Samples were collected for further characterization and analysis.

### GC-MS analysis

The Clarus 680 GC was used in the analysis employed a fused silica column, packed with Elite-5MS (5% biphenyl 95% dimethylpolysiloxane, 30 m × 0.25 mm ID × 250 μm df) and the components were separated using Helium as carrier gas at a constant flow of 1 ml/min. The injector temperature was set at 260 °C during the chromatographic run. The 1 μL of extract sample injected into the instrument the oven temperature was as follows: 60 °C (2 min); followed by 300 °C at the rate of 10 °C min^−1^; and 300 °C, where it was held for 6 min. The mass detector conditions were: transfer line temperature 240 °C; ion source temperature 240 °C; and ionization mode electron impact at 70 eV, a scan time 0.2 sec and scan interval of 0.1 sec. The fragments from 40 to 600 Da. The spectrums of the components were compared with the database of spectrum of known components stored in the GC-MS NIST (2008) library.

### Characterization

The crystalline behaviour and structural properties of the products were identified by XRD with CuKα1 (λ = 1.542 Å) radiation in the range of (2θ) from 20 to 80 °C at room temperature (Rigaku Smart Lab, Automated Multipurpose X-ray diffractometer, Japan). FTIR spectrophotometer was investigated with a JASCO FTIR- 460 plus spectrophotometer, Japan, to evaluate the surface chemistry of nanostructured materials. TEM and SAED was carried out by (Jeol/JEM 2100 model, USA, operating at 200 kV) to characterize the morphologies, size and the crystallinity and preferential orientation of the materials. The elemental analyses were executed by energy dispersive X-ray spectrophotometer (EDX), Bruker eAXS X flash X-Ray Detector. The UV-Vis spectroscopy was measured by SA 165 Diode array spectrophotometers to determine the band gap. XPS spectra of the samples were obtained using an ESCALAB 250Xi, Thermo Scientific instrument.

### Antimicrobial activity

The MIC of the ZnO-Ag NCs was evaluated according to the British Society for Antimicrobial Chemotherapy (BSAC) guidelines (Andrews 2001) by the microbroth dilution method^14^. The selective gram-negative and gram-positive bacteria (*E*. *coli* and *S*. *aureus*) were applied to assess the antibacterial potency of ZnO-Ag NCs with different concentration^57^. The bacteria were sub cultured in Luria broth at 37 °C overnight, and growth of the bacteria was evaluated with turbidity of Macfarland standard No. 0.5. The MIC and MKC of as-prepared samples were determined after 24 hours^58^. The assay was performed in three sets.

Disc diffusion method was carried out to evaluate the antimicrobial activity of prepared samples against *E*. *coli* and *S*. *aureus* on nutrient agar plate^59^. Fresh overnight cultures were utilized as a working bacterial suspension in Luria broth (concentration of 108 ml^−1^ of each bacteria). The antimicrobial activity was performed by spreading about 100 mL [108 CFU ml^−1^] of each bacterial culture on the agar surface by a sterile glass spreader. The discs with 6 mm diameter were placed on the NA plate. Antibiotics Gentamicin was used as positive control and a filter paper disc without the coatings was used as a control. Then the plates were incubated at 37 °C for 24 hours. The antibacterial activity was assessed by determining the diameter of the zone of inhibition against the bacteria.

### *In vitro* antiradical activity: DPPH method

The Free radical scavenging potency of ZnO-Ag NCs was performed with particular amendment as published recently^40^. One percent DPPH was applied as a source of free radical to evaluate the antiradical potency of as-prepared samples. ZnO-Ag NCs with various amounts (0.625 to10 mg/ml) were prepared and after that, 1.0 ml of prepared NCs and 1.0 ml of DPPH were mixed in the dark for 20 min. Lastly, the samples were centrifuged at 14,000 rpm for 7 min and the clear upper layer was analysed at ~517 nm using spectrophotometer. Three sets of experiments were performed and the average results were considered as a final outcome. The percentage of DPPH scavenging ability was computed as:

$${\rm{ \% }}\,DPPH\,antiradical\,potency=[(Ab{s}_{c}-Ab{s}_{s})/Ab{s}_{c}]\times 100$$

where Abs_s_ is the absorption of DPPH in the presence of sample and Abs_c_ is Absorption of DPPH in the absence of Sample. The DPPH antiradical scavenging of ascorbic acid was examined as a standard for comparison.

### Cytotoxicity assay (Haemolysis)

Preliminary evaluation of *in-vitro* biocompatibility of the ZnO-Ag NCs was carried out with haemolysis test, according to the ASTM standard E2524-8 which is the standard for investigation of hemolytic potency of NPs^60^. Haemolysis is damage of erythrocyte membrane and release of haemoglobin into the plasma. Haemolysis assay is a authentic measurement for assessing the biocompatibility of nanostructured materials^52^. The haemolysis activity experiment was conducted against ZnO-Ag NCs. 1 ml heparin as an anticoagulant was added to 10 ml Chicken blood mixed, centrifuged and then washed with physiological saline. 1 ml of the prepared sample and 9 ml of 1× DPBS was mixed. ZnO-Ag NCs with various amount (0.312–20 µg/mL) was sonicated and added to the prepared blood. After 2 hours incubation at room temperature, the samples were centrifuged. The supernatant was analysed using spectrophotometer and read at 541 nm. Positive and negative control samples were provided by using ultrapure water and blood into the saline, respectively. The following formula was used to calculate haemolysis percentage^40^

$${\rm{ \% }}\,{\rm{H}}{\rm{a}}{\rm{e}}{\rm{m}}{\rm{o}}{\rm{l}}{\rm{y}}{\rm{s}}{\rm{i}}{\rm{s}}=[({{\rm{A}}{\rm{b}}{\rm{s}}}_{{\rm{S}}{\rm{a}}{\rm{m}}{\rm{p}}{\rm{l}}{\rm{e}}}\,{\textstyle \text{-}}\,{{\rm{A}}{\rm{b}}{\rm{s}}}_{-{\rm{v}}{\rm{e}}})/({{\rm{A}}{\rm{b}}{\rm{s}}}_{+{\rm{v}}{\rm{e}}{\rm{C}}{\rm{o}}{\rm{n}}{\rm{t}}{\rm{r}}{\rm{o}}{\rm{l}}}\,{\textstyle \text{-}}\,{{\rm{A}}{\rm{b}}{\rm{s}}}_{-{\rm{v}}{\rm{e}}{\rm{C}}{\rm{o}}{\rm{n}}{\rm{t}}{\rm{r}}{\rm{o}}{\rm{l}}})]\times 100$$

where Abs_sample_ is absorbance of sample, Abs_+ve_ control is absorbance of positive control and Abs_−ve_ control is absorbance of negative control.

### Photocatalytic studies

Catalytic properties of the as-synthesized ZnO-Ag NCs was assessed by degradation of phenol under natural sunlight^61,62^. 60 mg/L catalyst and 0.8 ml of 20 mg/L phenol were mixed. The above suspension was continuously stirred with the help of magnetic stirrer in the dark for 1 hour to achieve adsorption equilibrium among the dye molecule and catalyst surface. After the elapse of a period of time, 5 ml of the aliquots of mixture were collected, filtered and centrifuged at 4000 rpm for 10 minutes. The samples were examined at 553 nm using UV Vis spectrophotometer.

Dye degradation efficiency was calculated by the following formula:

$${\rm{ \% }}\,{\rm{D}}{\rm{e}}{\rm{g}}{\rm{r}}{\rm{a}}{\rm{d}}{\rm{a}}{\rm{t}}{\rm{i}}{\rm{o}}{\rm{n}}=[({{\rm{A}}{\rm{b}}{\rm{s}}}_{0}-{{\rm{A}}{\rm{b}}{\rm{s}}}_{{\rm{t}}})/{{\rm{A}}{\rm{b}}{\rm{s}}}_{0}]\times 100$$

where Abs_0_ is initial absorbance and Abs_t_ is an absorbance at ‘t’ time.

### Mechanism for synthesis of Ag-ZnO NCs using *T*. *vulgaris* leaf extract

The following Equations represent the crystal nucleation and crystal growth steps for bio-hydrothermal synthesis of ZnO-Ag NCs.

$$\begin{array}{lll}{\mathrm{Zn}(\mathrm{NO}}_{3}{)}_{2}.6{{\rm{H}}}_{2}{\rm{O}}+2{\rm{NaOH}} &  & {{\rm{Zn}}}^{2+}{({\rm{OH}})}_{{\bf{2}}}^{-}+2\,{\mathrm{Na}(\mathrm{NO})}_{3}+{{\rm{6H}}}_{2}{\rm{O}}\\ + & \underrightarrow{{\rm{Leaf}}\,{\rm{extract}}} & \\ {\mathrm{Ag}(\mathrm{NO})}_{3} &  & {{\rm{Ag}}}^{+}+{{{\rm{NO}}}_{3}}^{-}\end{array}\underrightarrow{\triangle }\,\,{\rm{ZnO}}-{\rm{Ag}}\,{\rm{NCs}}$$

**Figure 11:**
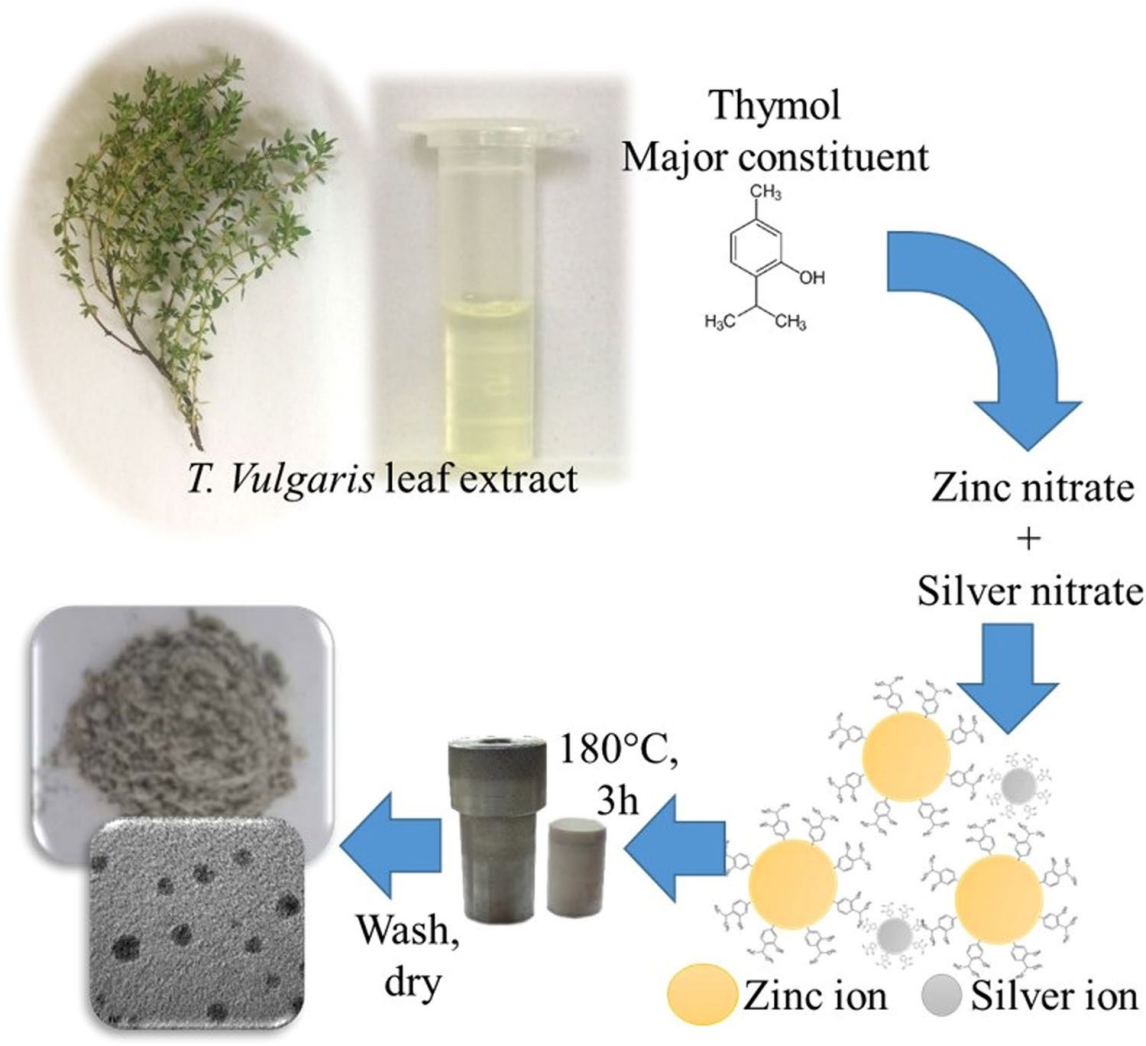
The proposed mechanism of ZnO-Ag NCs synthesis NCs.

The hypothetical description is illustrated in Fig. 11. Phenol derivatives (Thymol, Carvacrol) which are present in the leaf extract act as a size reducing during the synthesis of ZnO-Ag nanocomposite. The reducing agent (OH) in *T*. *vulgaris* leaf extract which was confirmed by FTIR reduce the Zn^2+^ and Ag+ ion to form ZnO and Ag NPs under the hydrothermal condition. Hence, reducing agents reacted with zinc nitrate and silver nitrate which reduced the interfacial tension of solvent and reducing the size by preventing the crystal growth. ZnO-Ag NCs were synthesized in presence of heat and pressure.

## Conclusions

To conclude, the present study established a facile eco-friendly method to synthesis ZnO-Ag NCs through a novel single step bio-hydrothemal method using *T*. *vulgaris* leaf extract. The phytochemical screening results verified the presence of phenol and flavonoid. Thymol is a type of phenolic monoterpenes, which is a substantial chemical components in the *T*. *vulgaris* leaf extract. Characterizations were accomplished using XRD, FTIR, UV-Visible, TEM, EDX, and SAED techniques, confirming the formation of ZnO-Ag NCs. TEM results confirm the existence of ~5 nm Ag NPs on the surface of ZnO NPs. The corresponding SAED pattern reveals the crystallinity and preferential orientation of the ZnO-Ag NCs pattern without any additional diffraction spots, which matches with the respective SAED and XRD results. Elemental composition of the nanocomposite demonstrates the presence of Ag (15.90%), ZnO (30.74%) and O (53.36%). XRD, HRTEM, SAED, XPS and EDX confirm the successful formation of ZnO-Ag nanocomposite. ZnO/Ag NCs illustrated synergistic and enhanced antimicrobial potency for both gram-positive and gram- negative bacteria attributed to the strong interaction between semiconductor ZnO and metallic Ag. Antioxidant activity of samples, as prepared above, was determined by DPPH method. The results show that the impact of ZnO NPs was improved by the synthesis of ZnO-Ag NCs thereby improving their antioxidant activity. Hemolysis results reveal that ZnO-Ag NCs caused the considerably higher degree of haemolysis, beyond 7% haemolysis at 10 µg/ml. Therefore, less than 10 µg/mL ZnO-Ag NCs does not damage the RBCs. The comparative photocatalytic degradation of ZnO NPs and ZnO-Ag NCs were investigated using phenol in the presence of visible light and the results ensure a drastic improvement of the photocatalytic activity of ZnO-Ag NCs. Hence, precisely designed ZnO-Ag NCS are anticipated to provide new avenues for the improvement of high-efficiency gas sensors, solar cells, biological application and food science. Moreover, further studies are required to assess the potential *in-vivo* cytotoxicity, biosafety and biocompatibility of such nanomaterials.
